# Can helicopter parenting be beneficial for parent–child relationships? A person-centered approach in the United States and South Korea

**DOI:** 10.3389/fpsyg.2023.1097348

**Published:** 2023-02-23

**Authors:** Woosang Hwang, Eunjoo Jung, Seonghee Kim, Narges Hadi

**Affiliations:** ^1^Department of Human Development and Family Sciences, Texas Tech University, Lubbock, TX, United States; ^2^Department of Human Development and Family Science, Syracuse University, Syracuse, NY, United States; ^3^Research Institute for Liberal Education, Yonsei University, Seoul, Republic of Korea

**Keywords:** helicopter parenting, college students, parent–child relationships, United States, South Korea, latent class analysis

## Abstract

**Introduction:**

We aimed to uncover latent classes of maternal and paternal helicopter parenting among American and Korean college students and to examine whether latent classes of maternal and paternal helicopter parenting are associated with parent– child relationships.

**Methods:**

We conducted three-step latent class analyses using five helicopter parenting indicators for 433 mother–child and 401 father-child groups in the United States and 207 mother–child and 195 father-child groups in South Korea.

**Results:**

We identified the same three helicopter parenting latent classes (*strong*, *weak*, and *managed*) in mother–child and father-child groups in the United States and Korea. In addition, we found that American and Korean college students in *strong* and *managed* helicopter parenting latent classes reported better parent– child relationships than those in the *weak* class regardless of parents’ gender.

**Discussion:**

Our findings indicate that helicopter parenting is multidimensional and can be similarly interpreted by college students in Western and Eastern societies. Further, our findings suggest that helicopter parenting could be helpful for college students to establish an intimate relationship with their parents.

## Introduction

The quality of relationships with parents continues to be of critical importance in the lives of young adults (Nelson and Padilla-Walker, 2013). Past and future interrelated experiences that parents and children build together serve as the basis for parent–child relationships (Özmete and Pak, 2022). A close parent–child relationship is a strong predictor of the well-being of young adult children (Van Wel et al., 2000; Hadiwijaya et al., 2020). Studies have identified children’s characteristics associated with parent–child relationships (e.g., communication and trauma experiences; Gillespie, 2020; Cui et al., 2021; Özmete and Pak, 2022). Others investigated contextual factors in relation to parent–child relationships (e.g., marital status, living arrangement, and marital conflict; Riggio and Valenzuela, 2011; Lander et al., 2013; Gillespie, 2020). Studies of families through the lens of family systems perspectives (Barnes and Olson, 1985; Olson, 2000) stress the importance of considering parenting behaviors for young adults as well (Segrin et al., 2012; Kwon et al., 2016; Dumont, 2021). However, past research has rarely examined the association between today’s prevalent over-parenting behaviors, i.e., helicopter parenting, with parent–child relationship outcomes (Nelson et al., 2021). Building relational competencies including parent–child relationships is a salient developmental task in emerging adulthood (Cui et al., 2022). Hence, it is important to study how helicopter parenting is related to parent–child relationships in families with young adults (Cui et al., 2022).

Culture has an immense influence on parenting practices and family values in any country. Families from Eastern and Western cultures are likely to have different parenting beliefs and practices (Harkness and Super, 1992; Raghavan et al., 2010; Cui et al., 2019). Prior work on helicopter parenting has mostly focused on young adults and families in American settings. A recent systematic review of research on helicopter parenting illustrates the lack of cross-cultural studies in the field. About 84% of the reviewed studies (62 out of 74) in the past 20 years (2002–2021) were conducted with only American participants. Approximately 16% (12 out of the 74) studied other countries, and just four of them investigated two different cultures (Cui et al., 2022). As such, we have little understanding of the association between helicopter parenting and parent–child relationships across different cultures, especially from Eastern and Western countries where there are variations in values and norms (Cui et al., 2019).

Research on helicopter parenting has yielded a strong body of empirical evidence on the effects of parenting practices on young adults’ physical, psychological, and emotional well-being outcomes (e.g., Padilla-Walker and Nelson, 2012; Bradley-Geist and Olson-Buchanan, 2014; Segrin et al., 2015; Darlow et al., 2017; Kouros et al., 2017). These investigations—a variable-centered approach—utilize average levels of helicopter parenting as a singular feature and can provide useful information for understanding the mechanisms of parenting practices. Nonetheless, there have been quite mixed (positive, negative, or neutral) results on child outcomes. More recent studies using a person-centered approach, such as latent class analysis, have shown that helicopter parenting may not be a singular construct, but rather a complex and multidimensional construct (Hwang et al., 2022; Love et al., 2022). Therefore, in this study, we investigate the multidimensional construct of helicopter parenting in emerging adulthood between the United States (United States hereafter) and South Korea (Korea hereafter). Asian countries including Korea, Japan, and China have a strong emphasis on parental authority and intergenerational harmony in Confucian cultures, and parents, in general, are highly involved in children’s social and academic lives which would be similar to the concept of helicopter parenting. Therefore, Korea has been selected in this study as a country that is reflective of traditional Confucian norms of parent–child relationships in Asian Cultures (Lee and Kang, 2018; Hwang et al., 2022). Additionally, we examine whether helicopter parenting latent classes are associated with parent–child relationships across the United States and Korean cultural contexts.

## Literature review

### Helicopter parenting

Helicopter parenting is characterized as the overly involved and excessively controlling behaviors of parents who intervene in their adult children’s daily functioning and social lives (Howard et al., 2020; Padilla-Walker et al., 2021). This parenting practice is usually regarded as developmentally inappropriate for young adults (Segrin et al., 2012; Rote et al., 2020) and is a variation of authoritarian parenting, which is highly controlling (Baumrind, 1971). Yet, researchers also shared that helicopter parenting may stem from parents’ well-intentioned support and concern for their children’s success and well-being and may not be inherently negative in nature (Fingerman, 2017; Love et al., 2020; Cui et al., 2022). Many studies have documented the adverse role of helicopter parenting on young adults’ outcomes including depression, psychological well-being, peer relationship development, and self-confidence (e.g., Darlow et al., 2017; Luebbe et al., 2018, Schiffrin et al., 2019). Others found that helicopter parenting is not that detrimental to young adults’ success and well-being (Padilla-Walker and Nelson, 2012; Earle and LaBrie, 2016). Yet, still others have shown that helicopter parenting is not directly related to adult children’s emotional well-being outcomes (Fingerman et al., 2012; Kwon et al., 2016). As such, the research findings on the role of helicopter parenting in young adults’ outcomes are quite mixed. This may be an indication that treating helicopter parenting as a unidimensional and singular construct may not fully capture the complex nature of parental behaviors for young adults.

A handful of studies have suggested that helicopter parenting is a multidimensional construct with different profiles (classes) that may vary in their influence on young adults’ outcomes (Luebbe et al., 2018; Hwang et al., 2022; Love et al., 2022). That is, helicopter parenting may not be just one factor (unidimensional construct) such as “helicopter parenting” (Padilla-Walker and Nelson, 2012; Schiffrin et al., 2014) but consists of several factors (multidimensional construct) that encompass different levels of parental involvement and varied parenting behaviors in regard to children’s lives (Segrin et al., 2012; Luebbe et al., 2018). For instance, overparenting (interchangeably used with helicopter parenting) has four domains—personal/social, academic/career, health, and financial—and each domain has different associations with young adults’ emotional dysregulation and problematic internet use outcomes among college students (Love et al., 2022; Schiffrin and Liss, 2022). Another study with college students showed three specific domains of helicopter parenting: information-seeking, direct intervention, and autonomy-limiting (Luebbe et al., 2018). While they found that the autonomy limiting dimension has a similar pattern as the helicopter parenting general factor, the other dimensions—information seeking and direct intervention—were selectively related or unrelated to other parenting factors. Another study showed that helicopter parenting is multidimensional, and the researchers identified three latent classes of helicopter parenting in relation to college students’ game and social media addictive behaviors—strong, strong but weak direct intervention, and weak, in both Eastern and Western countries, i.e., China and the United States (Hwang et al., 2022). The authors found one more latent class with American college students, weak but strong academic management, but that latent class was not found in the Chinese college student group. Building on these studies, it is necessary to investigate the multidimensional construct of helicopter parenting with college students across Eastern and Western countries to understand how to support young adults for their well-being and successful transition to adulthood.

### Helicopter parenting and parent–child relationship

Families and parental behaviors critically contribute to children’s social and emotional outcomes (Cui et al., 2022). Parent–child relationships that families have developed and evolved over the years in their family system continue to exert influence on young adults’ well-being and social life. Young adults who experienced positive parenting from their parents tend to have more positive relationships with other people in their adulthood. And those who have positive relationships with their parents tend to demonstrate better well-being than those who have less positive relationships (Dalton III et al., 2006). Family systems theory suggests that to fully understand individuals, the functioning mechanisms within the family system should be considered (Lander et al., 2013). Families develop healthy relationships when family members’ developmental needs and features are respected and honored (Olson, 2000). This would mean that families who keep balanced relationships, rather than excessively controlling relationships, would have greater family satisfaction and positive communication among family members (Barnes and Olson, 1985; Segrin et al., 2012). Hence, from the family systems perspective, helicopter parenting may not address the changing development needs of young adult children who need to make their own decisions, solve their own problems, and be autonomous in their daily and social lives.

While some children may consider their parents’ helicopter parenting as a daily and acceptable practice, others may not like that type of micromanaging and overinvolvement of their parents (Segrin et al., 2015; Su et al., 2021). Segrin et al. (2012) also showed that overparenting is related to low quality parent–child communication. Considering that young adults should explore their life options, be autonomous, and need to solve their own problems, adult children who have parents classified as strong helicopter parents are likely to show a less positive parent–child relationship. However, the multidimensionality of helicopter parenting patterns in relation to the parent–child relationship has not been tested thus far. Therefore, in this study, we investigate whether a latent class of helicopter parenting exists and, if so, how different patterns of helicopter parenting are related to parent–child relationships across cultures. Studies have suggested that helicopter parenting lies on a continuum from a weak helicopter parenting latent class to a strong helicopter parenting latent class and a distinctive natured helicopter parenting class (Luebbe et al., 2018; Hwang et al., 2022; Love et al., 2022).

Studies have shown that mothers and fathers have different parenting styles and interactions with their children. In general, mothers tend to have closer relationships with their children and provide more support, whereas fathers are more likely to have a distant and detached relationship with their children (Hosley and Montemayor, 1997; Kwon et al., 2017). Also, mothers usually provide warmer and more supportive parenting than fathers, and fathers are frequently engaged in a more directional parenting style driven by results (Bradley-Geist and Olson-Buchanan, 2014; Buchanan et al., 2016). Therefore, it is important to consider whether maternal and paternal helicopter parenting approaches have different latent classes in relation to the parent–child relationship. Although the relationship between latent classes of helicopter parenting and child outcomes in the family context has gained increased attention, no studies to date have investigated how maternal and paternal helicopter parenting are related to the parent–child relationship in families across Eastern and Western cultures. Exploring variations in mothers’ and fathers’ helicopter parenting may allow us to understand the familial context in which parent–child relationships are developed.

### Cultural contexts: The United States and South Korea

Children from different cultures have different perceptions and understanding of the parenting practices that they experience (Chao and Tseng, 2002; Bornstein and Cheah, 2006). Helicopter parenting and parent–child relationships across different cultures would have different patterns as well. Helicopter parenting has been studied mostly in the United States context (e.g., Reed et al., 2016; Cook, 2020; Cui et al., 2022) with a few cross-cultural studies that investigated the role of helicopter parenting in child well-being and academic outcomes in America, China, Finland, or Korea (Chao and Tseng, 2002; Cui et al., 2019; Jung et al., 2019).

Family is important in both Eastern and Western cultures, although there might be variations according to societal norms and values in the society. In Western cultures, individualism is more highly valued; in Eastern countries, collectivism and familism are more valued. Whereas people in Western countries tend to focus more on the independence and autonomy of their children, people in Eastern countries tend to sacrifice themselves for the common good and congenial relations with others and families (Chao and Tseng, 2002; Nelson et al., 2011). Furthermore, in Western cultures, parents tend to show their support for their children by being resourceful (Hill et al., 2004). When parents in the United States demonstrate over-involvement and excessive interference in their adult children’s lives, American adult children may develop adverse parent–child relationships due to their cultural background and their upbringing (Nelson et al., 2011). Yet, in Eastern cultures, helicopter parenting may be interpreted as a continuation of the parenting practice that they experienced as they were growing up, and adult children may interpret helicopter parenting differently (Chao and Tseng, 2002).

The helicopter parenting phenomenon has become prevalent across different cultures. Considering the influence of cultures in family processes, it is intriguing to examine whether helicopter parenting latent class would be distinct to a certain culture or would be observed across cultures, and whether helicopter parenting latent class has different or similar relations with parent–child relationships across different cultural contexts (Cui et al., 2019). As the cultural ecological perspective that emphasizes the importance of adaption to the environment in explaining the role of cultures elucidates (Steward, 1972), parenting behaviors would be better understood in specific cultural contexts (Garcia et al., 1996). Nonetheless, studies thus far have rarely examined such features across Eastern and Western countries. In addition, while there have been a handful of studies that investigated the cultural context of American settings (e.g., Cook, 2020; Room et al., 2020), less is known about the cultural context of Korea. Hence, a more in-depth understanding of Korean culture is warranted.

### Helicopter parenting studies in Korea

Considering the Korean cultural norm is patriarchal Confucianism, helicopter parenting is often observed in Korean society. This is linked to the cultural tendency to regard parental control over their children as natural. Through this, many emerging adults are likely to take parental care for granted, and it can be said that independence from parents is delayed (Choi, 2015). With this circumstance, the literature on helicopter parenting has been mainly conducted to examine the effect of psycho-emotional factors on undergraduate students (Park and Jin, 2021). For example, based on previous studies conducted in Western countries, studies on the association between helicopter parenting and depression have been conducted in Korea (Kim and Park, 2019). In addition, college students who perceived higher levels of helicopter parenting tended to have a lower number of children in the future (Yoo, 2014).

Despite the negative effects on psychological perspectives, helicopter parenting has a positive influence on Korean emerging adults’ transition to adulthood (Lee et al., 2014). This is because Korean emerging adults who were aware of helicopter parenting perceived that they received financial assistance and psychological stability from their parents because of emotional closeness. In a similar vein, there are a few studies indicating that helicopter parenting is associated with intimacy. For instance, the level of intimacy with parents is high among emerging adults who are aware of helicopter parenting (Choi, 2015). A study focused on the association between helicopter parenting and intimacy between adult children and parents reported that a higher level of perceived helicopter parenting led to a higher degree of intimacy (Kang and Lee, 2017). Helicopter parenting was also associated with higher levels of intimacy goals in dating (Kim, 2022).

When it comes to gender, Chun (2021) found that college students tended to perceive higher levels of maternal helicopter parenting than paternal ones. Additionally, the influence of maternal helicopter parenting on autonomy was greater in male college students than in female ones. Also, paternal helicopter parenting had an effect on self-evaluation only in male college students (Ahn and Seol, 2020). That being said, Ahn and Seol’s (2020) study reported that there were no differences in the perception of helicopter parenting itself between male and female college students. Taken together, empirical evidence of helicopter parenting on the parent–child relationship in the Korean context is lacking and inconclusive.

### The current study

To address the gap in the literature, we aimed to investigate the multidimensional construct of helicopter parenting in emerging adulthood in the United States and Korea. In addition, we aimed to examine whether helicopter parenting latent classes are associated with the parent–child relationship across American and Korean cultural contexts. Our research questions are as follows: Does helicopter parenting relate to parent–child relationships? How the association between helicopter parenting and parent–child relationships differs between American and Korean college students? We hypothesized that strong, weak, and other distinct helicopter parenting latent classes would be identified among college students. We also hypothesized that college students who were classified into a strong helicopter parenting latent class would report lower parent–child relationships than those who were classified into a weak helicopter parenting latent class. Additionally, we explored whether the above associations differ between maternal and paternal helicopter parenting and between the United States and Korea.

## Methods

### Participants

After Institutional Review Board approval, college students were recruited from one university in the United States and one university in Korea between March 2017 and February 2018. The two universities were selected based on their similar backgrounds (mid-sized and private universities in suburban areas) for comparative studies (Van De Vijver and Leung, 1997). The researchers visited undergraduate classes (mainly social science and liberal arts courses) and introduced the aim of the study to college students. Students who agreed to participate in the study completed the questionnaire and consent form and received extra credits as compensation. In total, 441 American students and 217 Korean students completed the survey. We constructed 433 mother–child and 401 father-child groups from the American student sample and 207 mother–child and 195 father-child groups from the Korean student sample.

### Measures

We used the translate-back translate method (Van De Vijver and Leung, 1997) to secure equivalent forms of measures. Prior to data collection, all the measures were pretested with college students in the United States and Korea.

#### Helicopter parenting

College students’ perceived maternal and paternal helicopter parenting were separately measured using five items of the Helicopter Parenting Scale (Padilla-Walker and Nelson, 2012). An example item was “My mother (father) makes important decisions for me.” Response options ranged from (1) *not at all* to (5) *a lot*. High scores represented greater helicopter parenting. Reliability scores were 0.78 and 0.77 for mother–child and father-child groups in the United States and 0.77 for mother–child and father-child groups in South Korea.

#### Parent–child relationship

Mother–child and father-child relationships were separately measured using five items of the Affectual Solidarity Scale (Mangen et al., 1988). An example item was “Taking everything into consideration, how close do you feel the relationship between you and your mother (father) is at this point in your life?” Response options ranged from (1) *not at all well* to (6) *extremely well*. High scores represented closer parent–child relationships. Reliability scores were 0.92 and 0.94 for mother–child and father-child groups in the United States and 0.87 and 0.94 for mother–child and father-child groups in South Korea.

#### Control variables

We used college students’ age, gender (0 = *male*, 1 = *female*), biological parent–child relations (0 = *stepparent*, 1 = *biological parent*), parents’ educational backgrounds (1 = *high school or less*, 4 = *Master’s/Doctoral degree*), and parents’ income (1 = *under $20,000* in the United States and *under W20,000,000* in Korea, 6 = *$100,001 or more* in the United States and *W100,000,001 or more* in Korea) as control variables, which are related to the quality of parent–child relationships (Silverstein et al., 1997; Hwang et al., 2018). We used college students’ race (0 = *others*, 1 = *white*) as a control variable in the American student groups only because all college students in Korea were Korean.

### Analytic strategy

To test the first hypothesis, we conducted a latent class analysis using Latent Gold 6.0 (Vermunt and Magidson, 2021). Latent class analysis is a person-centered approach that enables us to identify unobserved subgroups of the population based on their responses to a chosen set of indicators (Nylund-Gibson and Choi, 2018). Given that the original helicopter parenting indicators were ordinal items, we initially conducted a latent profile analysis. However, we were not able to identify the best-fitting model. As such, dichotomizing indicators are recommended when ordinal items are highly skewed and cannot fully represent the range of responses (Vasilenko, 2022). We found that two items of maternal and paternal helicopter parenting (“My mother/father intervenes in setting disputes with my roommates or friends” and “My mother/father intervenes in solving problems with my employers or professors”) were skewed in mother–child and father-child groups in the United States and Korea. Therefore, we dichotomized five items of helicopter parenting into low (1 = *not at all*, 2 = *slightly*) and high (3 = *moderately*, 4 = *very*, 5 = *a lot*) categories and conducted a latent class analysis. To determine the optimal number of latent classes, the Bayesian Information Criterion (BIC), the Consistent Akaike Information Criterion (CAIC), and the Sample-Adjusted Bayesian Information Criterion (SABIC) were used. The latent class with the smallest BIC, CAIC, and SABIC values were utilized as the best-fitting model (Nylund-Gibson and Choi, 2018).

To test the second hypothesis, we used a three-step BCH latent class approach, a bias-adjusted method correcting for classification errors (Bolck et al., 2004). After identifying latent classes, college students were assigned a membership probability in each latent class. Next, multivariate regression analyses of the associations between class membership, parent–child relationships, and control variables were conducted, weighted by latent class probability membership. We used a full-information maximum likelihood estimation to account for missing values (Vermunt and Magidson, 2021).

## Results

### Descriptive analyses

Descriptive results regarding college students’ demographic characteristics and study variables are presented in Table 1. The mean age of American and Korean college students was 19 and 21 years old, respectively. Regarding gender, ~70% and 55% were female in American and Korean college students, respectively. In terms of parent–child relationships, both American and Korean college students reported that their relationships with mothers were closer than with their fathers.

**Table 1:** Descriptive results.

Variables	Range	United States				South Korea			
		Mother–child (*n* = 433)		Father-child (*n* = 401)		Mother–child (*n* = 207)		Father-child (*n* = 195)	
		*n* (%)	*M* (SD)	*n* (%)	*M* (SD)	*n* (%)	*M* (SD)	*n* (%)	*M* (SD)
Demographic characteristics									
Age	18–25		19.77 (1.25)		19.76 (1.26)		21.49 (1.92)		21.48 (1.92)
Gender									
Male		122 (28.2)		111 (27.7)		93 (44.9)		88 (45.1)	
Female		308 (71.1)		287 (71.6)		114 (55.1)		107 (54.9)	
Race									
White		270 (62.4)		260 (64.8)		-		-	
Others		162 (37.4)		140 (34.9)		-		-	
Family structure									
Biological mother/father		412 (95.2)		365 (91.0)		207 (100.0)		184 (94.4)	
Stepmother/father		21 (4.8)		36 (9.0)		0 (0.0)		11 (5.6)	
Parents’ education	1–4		2.72 (0.98)		2.69 (1.08)		2.18 (1.08)		2.61 (1.03)
Parents’ annual income	1–6		4.62 (1.58)		4.71 (1.55)		3.63 (1.42)		3.73 (1.38)
Helicopter parenting indicators									
Makes important decisions for me	1–5		2.66 (1.23)		2.30 (1.22)		3.43 (1.15)		3.20 (1.24)
Low category		204 (47.1)		234 (58.4)		43 (20.8)		60 (30.8)	
High category		229 (52.9)		167 (41.6)		164 (79.2)		135 (69.2)	
Intervenes in issues with friends	1–5		1.64 (1.01)^a^		1.39 (0.83)^a^		1.60 (0.91)^a^		1.42 (0.80)^a^
Low category		352 (81.3)		361 (90.0)		170 (82.1)		170 (87.2)	
High category		80 (18.5)		39 (9.7)		37 (17.9)		25 (12.8)	
Intervenes in issues with professors	1–5		1.76 (1.09)^a^		1.55 (0.96)^a^		1.52 (0.84)^a^		1.45 (0.82)^a^
Low category		342 (79.0)		342 (85.3)		177 (85.5)		175 (89.7)	
High category		91 (21.0)		59 (14.7)		30 (14.5)		20 (10.3)	
Solves crisis/problems	1–5		2.96 (1.17)		2.49 (1.23)		2.94 (1.14)		2.81 (1.23)
Low category		160 (37.0)		208 (51.9)		72 (34.8)		80 (41.0)	
High category		269 (62.1)		189 (47.1)		135 (65.2)		115 (59.0)	
Looks for jobs for me	1–5		2.75 (1.36)		2.41 (1.38)		2.46 (1.26)		2.49 (1.30)
Low category		191 (44.1)		220 (54.9)		112 (54.1)		99 (50.8)	
High category		242 (55.9)		181 (45.1)		95 (45.9)		96 (49.2)	
Parent–child relationships	1–6		4.92 (1.06)		4.53 (1.31)		4.50 (0.99)		4.03 (1.23)

a The absolute value of skewness exceeds 1.

### Identifying helicopter parenting latent classes

**Figure 1:**
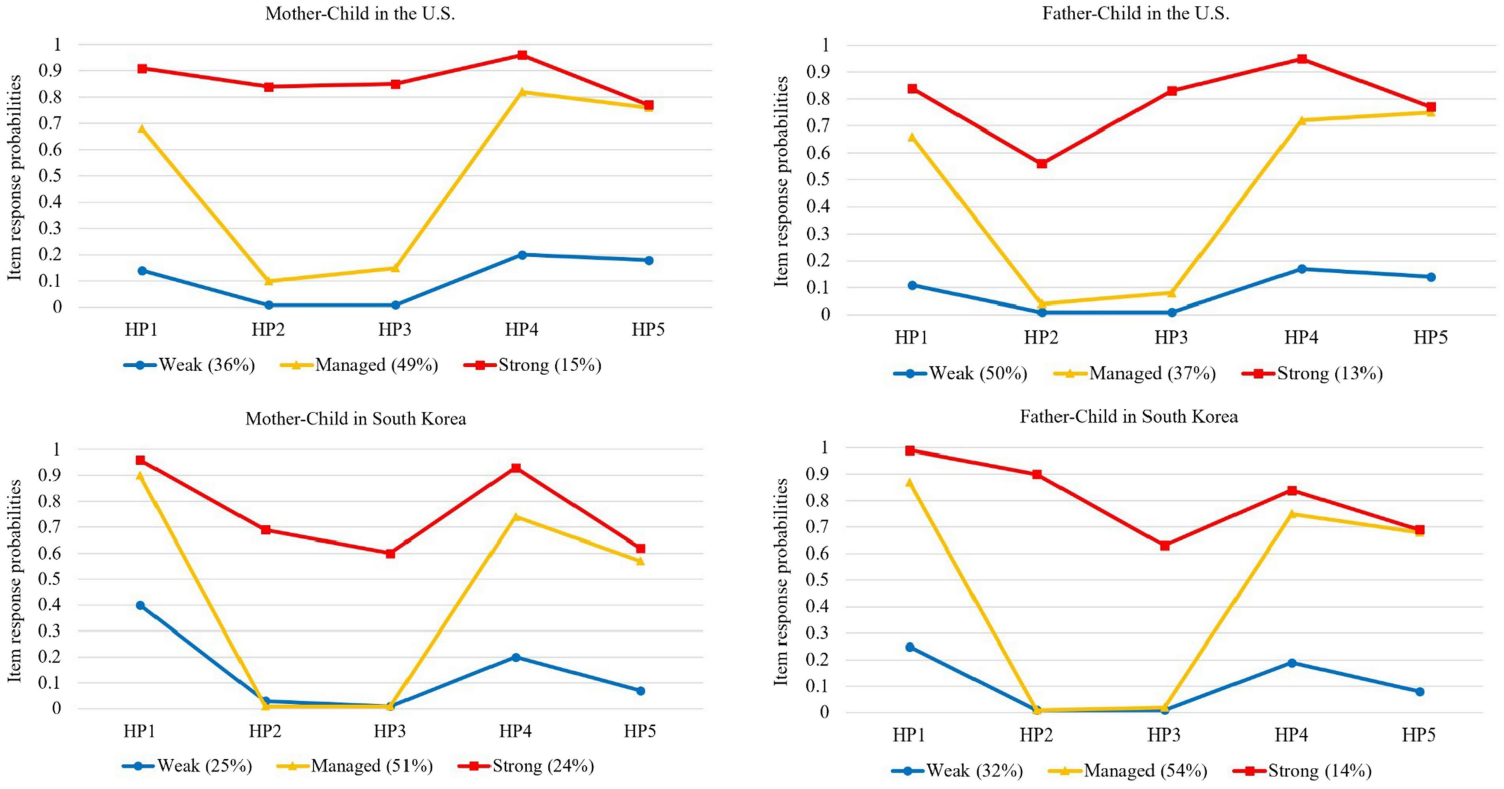
Item response and Latent class probabilities in mother–child and father-child groups in the United States and Korea. HP1 = “My mother/father makes important decisions for me (e.g., where I live, where I work, what classes I take).” HP2 = “My mother/father intervenes in settling disputes with my roommates or friends.” HP3 = My mother/father intervenes in solving problems with my employers or professors.” HP4 = My mother/father solves any crisis or problem I might have.” HP5 = My mother/father looks for jobs for me or tries to find other opportunities for me (e.g., internships, study abroad).

Results of latent class analysis are presented in Table 2. Three fit indicators suggested that the three-class model was the best-fitting model for mother–child and father-child groups in both American and Korean college students. Item response and latent class probabilities of three classes are presented in Figure 1. The first class was labeled *weak*. In this class, item response probabilities of all items were below 0.5. The second class was labeled *managed.* In this class, item response probabilities of three items (“Makes important decisions for me,” “Solves any crisis or problems I might have,” and “Looks for jobs for me or tries to find other opportunities for me”) were >0.5, but two items (Intervenes in setting disputes with my roommates or friends” and “Intervenes in solving problems with my employers or professors”) were below 0.5. The third class was labeled *strong*. In this class, item response probabilities of all items were more than 0.5.

**Table 2:** Latent class analysis statistics and fit indices.

Classes	United States						South Korea					
	Mother–child (*n* = 433)			Father-child (*n* = 401)			Mother–child (*n* = 207)			Father-child (*n* = 195)		
	BIC	AIC	SABIC	BIC	AIC	SABIC	BIC	AIC	SABIC	BIC	AIC	SABIC
1	2649.41	2629.05	2633.54	2266.82	2246.85	2250.95	1156.91	1140.25	1141.07	1079.70	1063.33	1063.86
2	2404.05	2359.27	2369.14	**2033.65**	1989.72	1998.75	**1096.60**	1059.94	1061.75	1008.31	972.31	973.47
3	**2386.42**	2317.21	**2332.47**	2035.48	**1967.58**	**1981.54**	1098.18	**1041.53**	**1044.32**	**995.20**	**939.56**	**941.34**
4	2407.43	**2313.80**	2334.44	2069.60	1977.74	1996.62	1124.68	1048.03	1051.81	1021.46	946.18	948.59
5	2440.90	2322.84	2348.87	2103.89	1988.07	2011.88	1155.75	1059.10	1063.87	1052.88	957.96	961.01
6	2474.73	2332.25	2363.65	2137.87	1998.09	2026.82	1187.31	1070.66	1076.41	1084.39	969.84	973.52

Bolded values indicate the best fit for each respective statistic. BIC, Bayesian Information Criterion; AIC, Consistent Akaike Information Criterion; SABIC, Sample-size Adjusted Bayesian Information Criterion.

### Associations between latent classes and parent–child relationships

Results of multivariate regression analyses are presented in Table 3 (reference group: *weak* class). We found that college students in *strong* and *managed* latent classes reported close mother–child and father-child relationships compared to those in the *weak* latent class in the United States and Korea. These results indicate that *managed* and *strong* types of helicopter parenting would be beneficial for college students’ perceived parent–child relationships regardless of parents’ gender and cultural contexts. We additionally conducted a paired comparison test to investigate whether parent–child relationships differ between managed and strong helicopter parenting latent classes (not presented in Table 3). However, parent–child relationships were not significantly different between *managed* and *strong* latent classes in mother–child and father-child groups in the United States and Korea.

**Table 3:** Association of latent classes with mother–child and father-child relationships.

Variable	United States		South Korea	
	Mother–child (*n* = 433)	Father-child (*n* = 401)	Mother–child (*n* = 207)	Father-child (*n* = 195)
	*b* (SE)	*b* (SE)	*b* (SE)	*b* (SE)
Latent classes (ref: weak)				
Managed	0.50 (0.16)**	0.63 (0.18)***	0.56 (0.26)*	0.90 (0.27)***
Strong	0.43 (0.16)**	0.78 (0.18)***	0.73 (0.25)**	1.22 (0.29)***
Control variables				
Age	0.07 (0.04)	−0.05 (0.05)	−0.00 (0.04)	0.01 (0.05)
Female (vs. male)	0.21 (0.10)*	−0.01 (0.12)	−0.05 (0.16)	−0.15 (0.22)
White (vs. others)	0.51 (0.11)***	0.53 (0.14)***	–	–
Stepparent (vs. biological parent)	−0.13 (0.23)	−0.63 (0.26)*	–^a^	−0.09 (0.39)
Parents’ education	0.01 (0.05)	0.01 (0.06)	0.11 (0.06)	−0.16 (0.08)
Parents’ income	0.04 (0.03)	0.13 (0.05)*	−0.03 (0.05)	0.09 (0.07)

*p < 0.05. **p < 0.01. ***p < 0.001. a=All mothers were biological mothers. Thus, this variable was excluded in the analysis.

## Discussion

In this study, we investigated whether helicopter parenting latent classes are associated with a parent–child relationship between American and Korean college students. The data supported the first hypothesis that two polar helicopter parenting classes (*weak* and *strong*) and one distinctly heterogeneous helicopter parenting class (*managed*) were equally identified in mother–child and father-child groups in the United States and Korea. The characteristic of the *managed* latent class is that parents are mainly involved in their children’s academic and life transition issues, but they are rarely involved in their children’s personal issues, such as conflict with friends, roommates, and professors. However, *weak* and *strong* latent classes represented low and high levels of helicopter parenting. This finding supports previous studies, which found that helicopter parenting can be multidimensional (Luebbe et al., 2018; Love et al., 2022).

Regarding the second hypothesis, contrary to our expectation, college students in the *strong* and *managed* helicopter parenting latent classes reported a closer parent–child relationship than those in the *weak* helicopter parenting latent class regardless of parents’ gender and cultural differences. We speculate that college students may perceive low levels of helicopter parenting as uninvolved or neglectful parenting. In this parenting style, parents are not interested in their young-adult children’s lives (Baumrind, 1971). Although helicopter parenting implies intrusiveness, it also reflects “increased contact, intimacy, and parental support” (Fingerman, 2017, p. 6). From the college students’ perspective, parental support is an important resource for their successful transition to adulthood. Therefore, it can be interpreted that parents’ indifference to their children’s needs and lives would be a risk factor for decreasing the relationship quality with their parents. However, we note that our finding does not indicate that helicopter parenting is an appropriate parenting style for parents with young-adult children. Related, Padilla-Walker and Nelson (2012) argued that characteristics of helicopter parenting (e.g., guidance and emotional support) would be related to young adult children’s positive feelings which may affect their quality of parent–child relationships. Therefore, future studies should examine the association between helicopter parenting and parent–child relationship in various contexts.

### Implications

The findings of this study highlight significant insights for family practitioners, parents, educators, and researchers attempting to understand the implications of helicopter parenting in emerging adulthood in different cultures. Both American and Korean college students with strong and managed helicopter parenting perceived a closer relationship with their parents compared to those in the weak helicopter parenting latent class. Thus, our results suggest that college students may not view helicopter parenting as extreme or invasive. This study informs parents, practitioners, and administrators on how various types of helicopter parenting might lead to different qualities of parent–child relationships.

Our findings also provide practical implications for higher education institutions. Some institutions are implementing a parental intervention to enhance parenting engagement during children’s transition to college (Love et al., 2020). To protect offspring from failures, helicopter parents might demonstrate behaviors such as parental investment, involvement, and care to take over their children’s responsibilities (Bradley-Geist and Olson-Buchanan, 2014). These behaviors might lead to emerging adult children’s success (Van Ingen et al., 2015). The results of this study help higher education institutions to know the degree to which parenting engagement might be useful. This study may also help them in implementing some parental programs to facilitate college students’ higher retention rates, academic success, and physical and mental health.

Additionally, the results of this study promote our understanding of the role of parenting during the transition to adulthood by illustrating the association between various types of helicopter parenting and parent–child relationships. Our results suggest that parental involvement might provide significant support for college students who are approaching issues of adult life. The transition to adulthood causes important affective distress due to reduced social support during this period. Therefore, family and mental health counselors should help adults to cope with those challenges by encouraging college students to maintain high-quality relationships with their parents (Nelson and Padilla-Walker, 2013).

Although the findings highlight the positive aspects of helicopter parenting in the parent–child relationship, there is much to be evaluated regarding the complex interactions between various types of helicopter parenting and parent–child relationships in emerging adulthood. The mixed effects of helicopter parenting on the parent–child relationship should further explored in future studies. Additionally, a longitudinal design is needed to explore how the different types of overparenting might influence and change the parent–child relationship over time. This study focused on just two universities from two cultures. Thus, future research also needs to extend the study to multiple universities from numerous cultures to effectively and comprehensively examine how various classes of helicopter parenting might be associated with parent–child relationships across different cultures.

### Limitations

This study has limitations. First, this study relied on college students’ reports of maternal and paternal helicopter parenting. Therefore, future studies should examine the association between helicopter parenting and parent–child relationships using dyadic data. Second, as we mentioned earlier, this study is a cross-sectional study. Therefore, we were not able to explain the causal association between helicopter parenting and parent–child relationships. For example, from the parents’ perspective, low quality parent–child relationships may lead to their uninvolved parenting style. It would be interesting to investigate whether the parent–child relationship is associated with stability and change of helicopter parenting latent classes over time. Third, our sample is not nationally representative, thus caution is needed if generalizing to other college students in the United States and South Korea.

## Conclusion and recommendation

Overall, this study highlights the multidimensional construct of overparenting and its associations with parent–child relationships in emerging adulthood in the United States and Korea. The findings illuminate that college students in strong and managed latent classes experienced a close relationship with their parents compared to those in the weak latent class in both cultures. This study provides also initial evidence that, regardless of gender and cultural contexts, the strong and managed types of helicopter parenting could be helpful for college students to establish a close relationship with their parents. This study could contribute to the literature by filling the gap between research regarding helicopter parenting and the parent–child relationship in families across Eastern and Western cultures. Exploring variations in mothers’ and fathers’ helicopter parenting added the knowledge of the familial context in which parent–child relationships were developed. This study also could complement the previous gap by providing practical implications for higher education institutions such as parental intervention to increase parenting engagement during children’s transition to college.

The findings of this study do not make any suggestions regarding best practices for the encouragement or discouragement of overparenting. We acknowledge that many studies have reported that helicopter Parenting has many negative effects on college students’ well-being (Jung et al., 2020; Cui et al., 2022). However, our findings provide evidence that parents’ support, care, involvement, and emotional support may be an important factor in a closer parent–child relationship for college students for American and Korean cultures. With this foundation, we suggest that helicopter parenting would be helpful for college students to establish an intimate relationship with their parents.

## Data availability statement

The raw data supporting the conclusions of this article will be made available by the authors, without undue reservation.

## Ethics statement

The studies involving human participants were reviewed and approved by Syracuse University, Yonsei University. The patients/participants provided their written informed consent to participate in this study.

## Author contributions

WH: designed and executed the study, analyzed data, and wrote the paper. EJ and SK: collaborated with data collection and wrote the paper. NH: wrote the paper. All authors contributed to the article and approved the submitted version.

## Funding

This work was supported by the Ministry of Education of the Republic of Korea and the National Research Foundation of Korea (NRF-2022S1A5C2A04093488).

## Conflict of interest

The authors declare that the research was conducted in the absence of any commercial or financial relationships that could be construed as a potential conflict of interest.

## Publisher’s note

All claims expressed in this article are solely those of the authors and do not necessarily represent those of their affiliated organizations, or those of the publisher, the editors and the reviewers. Any product that may be evaluated in this article, or claim that may be made by its manufacturer, is not guaranteed or endorsed by the publisher.

## Author disclaimer

This manuscript has not been published and is not under consideration for publication elsewhere. All the authors listed in the byline have agreed to the byline order.
